# Metal artifact reduction on cervical CT images by deep residual learning

**DOI:** 10.1186/s12938-018-0609-y

**Published:** 2018-11-27

**Authors:** Xia Huang, Jian Wang, Fan Tang, Tao Zhong, Yu Zhang

**Affiliations:** 1 School of Biomedical Engineering, Southern Medical University, Guangzhou, 510515 Guangdong China; 2 Guangdong Provincial Key Laboratory of Medical Image Processing, Southern Medical University, Guangzhou, 510515 Guangdong China

**Keywords:** Metal artifact reduction, Residual learning, Convolutional neural network, Cervical CT

## Abstract

**Background:**

Cervical cancer is the fifth most common cancer among women, which is the third leading cause of cancer death in women worldwide. Brachytherapy is the most effective treatment for cervical cancer. For brachytherapy, computed tomography (CT) imaging is necessary since it conveys tissue density information which can be used for dose planning. However, the metal artifacts caused by brachytherapy applicators remain a challenge for the automatic processing of image data for image-guided procedures or accurate dose calculations. Therefore, developing an effective metal artifact reduction (MAR) algorithm in cervical CT images is of high demand.

**Methods:**

A novel residual learning method based on convolutional neural network (RL-ARCNN) is proposed to reduce metal artifacts in cervical CT images. For MAR, a dataset is generated by simulating various metal artifacts in the first step, which will be applied to train the CNN. This dataset includes artifact-insert, artifact-free, and artifact-residual images. Numerous image patches are extracted from the dataset for training on deep residual learning artifact reduction based on CNN (RL-ARCNN). Afterwards, the trained model can be used for MAR on cervical CT images.

**Results:**

The proposed method provides a good MAR result with a PSNR of 38.09 on the test set of simulated artifact images. The PSNR of residual learning (38.09) is higher than that of ordinary learning (37.79) which shows that CNN-based residual images achieve favorable artifact reduction. Moreover, for a 512 × 512 image, the average removal artifact time is less than 1 s.

**Conclusions:**

The RL-ARCNN indicates that residual learning of CNN remarkably reduces metal artifacts and improves critical structure visualization and confidence of radiation oncologists in target delineation. Metal artifacts are eliminated efficiently free of sinogram data and complicated post-processing procedure.

## Background

Cervical cancer is the fifth most common cancer among women, which is the third leading cause of cancer death in women worldwide,with approximately five hundred thousand women developing this disease annually [1]. Clinically, brachytherapy is the most effective treatment for cervical cancer. For brachytherapy, computed tomography (CT) imaging is necessary since it conveys tissue density information which can be used for dose planning. During brachytherapy, afterloading devices with different types of metallic applicators are generally implanted inside the vaginal cavity of the patients with cervical cancer [2, 3]. However, these metallic implants have much higher attenuation coefficients than bones or soft tissue. The X-rays were heavily attenuated after passing through metal objects, resulting in only weak signals reaching the detector. In this situation, if the X-ray detector lacks a sufficient dynamic range in detecting the weak signal, there will be metal shadows in the raw projection data. These metal shadows will introduce streak artifacts, which can spread to nearby soft tissue regions in the reconstructed cervical CT images, obscuring the crucial diagnostic information of the tissues surrounding the metallic implants [4]. These metal artifacts caused by brachytherapy applicators remain a challenge for the automatic processing of image data for image-guided procedures or accurate dose calculations [5]. Therefore, developing an effective metal artifact reduction (MAR) algorithm in cervical CT images is of high demand.

A number of techniques have been proposed to reduce metallic artifact in CT images. These methods can generally be categorized into three major groups: (a) acquisition improvement, (b) sinogram completion methods and (c) model-based iterative algorithms. With acquisition improvement methods [6–8], increasing mAs and Kv will increase the number of photons, reducing noise, and narrow the profile of photon energies. Increased slice thickness will improve the signal to noise ratio, but can be associated with increased partial volume artifacts. Increasing the CT scale will improve the appearance of streak artifact. Dual Energy CT as a relatively new approach has brought about several advances in clinical CT interpretation, largely by improving the specificity of diagnostic information [6]. However, Dual Energy CT requires longer computational time in post-processing and has higher radiation dose compared with Single Energy CT. With the sinogram (or projection) completion methods [9–12], areas of data corrupted by the presence of the metal, are identified in the sinogram space. That data are then treated as missing and is replaced using interpolation routines based on the uncorrupted sinogram regions. CT image reconstruction, typically a filtered back projection (FBP) type algorithm, is then applied. A first example of a sinogram completion method applied to CT image MAR is the algorithm suggested by Kalender et al. [9]. A more advanced approach, the normalized metal artifact reduction (NMAR) algorithm, has been used in several studies [10]. In addition, to improve the edge information of surrounding bone structures, the application of a frequency split metal artifact reduction (FSMAR) algorithms was introduced by Meyer et al. [12]. However, full removal of projections performed by these approaches is associated with loss of information and inaccuracies in the estimated completion data may lead to additional artifacts in the reconstructed images or loss of spatial resolution. As an alternative approach to the sonogram completion methods, model-based iterative algorithms [13–15] work under the assumption that most artifacts arise because some data are missing or deviate from the model used for the data acquisition. Utilizing prior knowledge of the imaging physics, the measurement statistics and the image statistics, iterative reconstruction is applied to better interpret the measured projection data. The selective algebraic reconstruction technique (SART) [14] involves the use of an algebraic reconstruction to try to match the projection data to within the experimental error. In each SART iteration, an edge-preserving blur filter is applied to guide convergence to a smoother image form the large set of images consistent with the projection data. Metal detection technique (MDT) [14] is another model-based iterative algorithm. With MDT, forward projection is performed iteratively to replace detector measurements that involve metal. However, the performance of these methods is subject to the level of the accuracy of the physics models utilized and the prior knowledge of the shape and location of the metal objects. A brief summary of the existing MAR methods mentioned above is shown in Table 1.

**Table 1:** The differences between our proposed methods and other methods

Methods	Non-raw data	Non-post processing	Run time
*Acquisition improvement*			
Bamberget al. [6]	✓	✗	–
Schoeppel et al. [7]	✓	✗	–
Fabian et al. [6]	✓	✓	Long
*Sinogram completion*			
Kalender et al. [9]	✗	✓	–
Meyer et al. [10]	✗	✓	–
Roeske et al. [11]	✗	✓	–
*Iterative reconstruction*			
Xia et al. [13]	✗	✓	Long
Boas et al. [14].	✗	✓	Long
Aissa et al. [15]	✗	✓	Long
*Proposed*			
RL-ARCNN	✓	✓	< 1 s

Recently, deep learning methods have been successfully used in the fields of image processing and pattern recognition processes, such as image denoising [16, 17], image super- resolution [18, 19], and low-dose CT reconstruction [20–23]. Similarly, deep learning algorithms are also applied to metal artifacts reduction [24–28]. For examples, the first deep learning-based method used in MAR is introduced by Gjesteby et al. [28]..This method combined deep learning model with a state-of-the-art NMAR algorithm working in the reconstructed images to correct metal artifacts in critical image regions. The experiments demonstrated that deep learning model (DLM) can overcome the errors from the NMAR, and achieve better image quality. Park et al. [27] used U-net to repair inconsistent sinogram by removing the primary metal-induced beam-hardening factors along the metal trace in the sinogram. Zhang et al. [29] developed a DLM based open MAR framework to remove metal artifacts of CT images.

Many methods mentioned above have been proposed to remove image artifacts and recover information about underlying structures. However, most existing works were used in dental CBCT [30, 31], male pelvic CT [32] and only several works [7, 11, 13, 33] were studied in cervical CT, but reported their results obtained on different databases and it makes the direct comparison very difficult. Therefore, there is still no a general approach to well removes metal artifacts in cervical CT images. Nowadays, MRI based image guided brachytherapy has been discussed by many researchers, since MRI has higher soft tissue resolution and no radiation dose. However, MRI based cervix cancer brachytherapy has three main limitations: the presence of intrinsic image distortion, the lack of attenuation coefficient information needed for the correction of tissue inhomogeneities in dosimetry calculations, and the absence of bone information for portal verification. At present, CT based cervix cancer brachytherapy is still the main treatment modality for cervical cancer.

In this work, we propose a deep learning-based cervical cancer metal artifact reduction method in cervical CT images. Our contribution is threefold: (i) A novel RL-ARCNN is proposed to reduce metal artifacts in cervical CT images. The proposed model is designed to predict residual images (the difference between artifact image and artifact-free image) rather than to directly output the reduced artifact image, which is the key difference between metal artifacts and clean images. (ii) We generate simulated artifact cervical CT images to train and verify the proposed model quantitatively. (iii) Verified on clinical artifact cervical CT images, our method illustrates preferable performance. The rest of the paper is organized as follows. “Methods” section describes the data generation and the proposed convolutional neural network (CNN) model and its training procedure, followed by the “Experimental results” section. “Discussion” and “Conclusion” are given in last two sections, respectively.

## Methods

### Data generation

A simulated artifact cervical CT image dataset is created for RL-ARCNN training. In this dataset, artifact-free, metal artifact (artifact-insert), and artifact-residual images (the difference between artifact-free and artifact-insert images) are generated. The artifact-free images are the CT images (these images are without artifact) from cervical cancer patients obtained before brachytherapy, and artifact-insert images are generated by simulating metal implants.

**Fig. 1:**
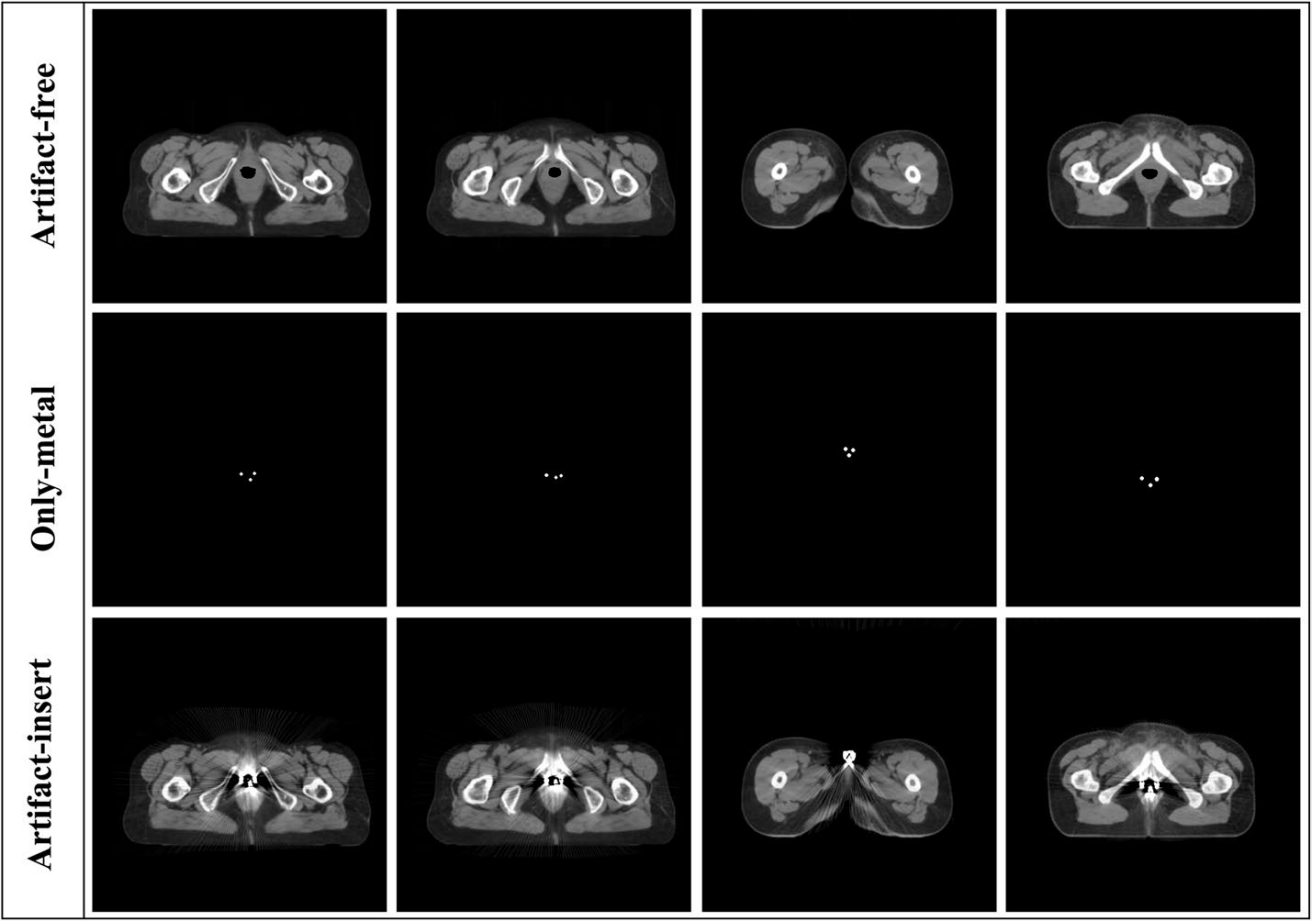
The example of the images simulation. The three rows are artifact-free images, only-metal images and artifact-insert images, respectively

The shape, size, and position in the CT images of metal implant are manually simulated and stored as small binary images. The algorithm proposed by Zhang et al. [29] is performed to generate artifact-insert images. The images are segmented into metal, bone, and water equivalent by soft-threshold-based weighting [34] and set to corresponding attenuation coefficients. Subsequently, a forward projection approach is employed to reconstruct the artifact-insert images. Figure 1 shows an example of the generated artifact images.

### Deep CNN training

We systematically present the MAR approach based on RL-ARCNN. In general, deep CNN model training for a specific task involves two steps: (i) network architecture design and (ii) model learning from training data. In this work, a modified VGG architecture [35] is used, and a similar architecture has been employed for image denoising and restoration [36–39].

#### Architecture

**Fig. 2:**
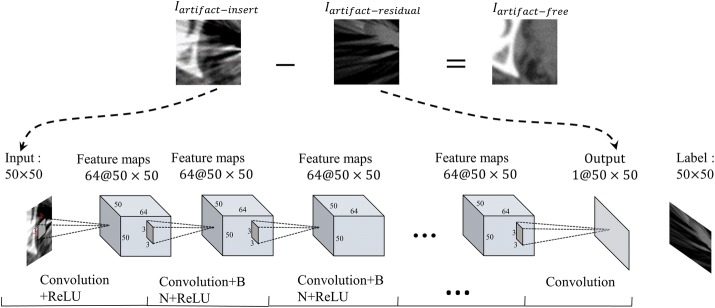
The architecture of convolutional neural network for metal artifacts reduction

Given the RL-ARCNN with depth D, three types of layers are designed, as shown in Fig. 2. The input layer is a 50 × 50 image patch generated by extracting a rectangular region from input images. For the first layer, a total of 64 filters with 3 × 3 × 1 size are used to generate 64 feature maps that involve detailed local textures and capture considerable relevant edge information, then the rectified linear units utilized (ReLU) for nonlinearity defined as ${\text{ReLU}} = { \hbox{max} }\left( {0,{\text{x}}} \right)$. In the first layer, the output after convolution can be formulated as:

1 $$C_{1} \left( p \right) = ReLU\left( {W_{0} *p + b_{0} } \right)$$

where *W*_0_ and *b*_0_ denote the weights and biases respectively, * means convolution, *p* is extracted image patch from input images. *C*_1_(*p*) means new feature maps based on the first layer’s output.

Subsequently, for 2 ~ D-1 layers are placed to extract the local features from feature maps by convolution, which is performed between the previous layers and a series of filters. The number of filters is set to 64 with 3 × 3 × 1 kernels. The convolutional layer provides nonlinear mapping from low-level to high-level representation of images. Zero padding is performed before convolution to ensure that each feature map of the middle layers possesses the same size as the input image. Subsequently, an element-wise non-linear activation function is applied on the output of each convolutional layer, which significantly affects the speed of convergence. In our work, ReLU activation function is employed [40], in which the low-negative slope coefficient is equal to the task. Batch normalization is added between convolution and ReLU as it can achieve fast training, good performance, and low sensitivity to initialization [41]. In the 2 ~ D-1 layers, the output after convolution and batch normalization (BN) can be formulated as:

2 $$C_{d} \left( p \right) = ReLU\left( {W_{d - 1} *C_{d - 1} \left( p \right) + b_{d - 1} } \right)\quad {\text{d}} = 2 \ldots {\text{D}} - 1$$

where *W*_*d*-1_ and *b*_*d*-1_ denote the weights and biases in the d^th^ layers, respectively, * means convolution, *C*_*d*_(*p*) generates new feature maps based on the (*d* − 1)^th^ layer’s output.

For the last layer, 1 filter with a 3 × 3×64 size is used to generate the output. Then, we have:

3 $$C_{D} \left( p \right) = W_{D - 1} *C_{D - 1} \left( p \right) + b_{D - 1}$$

Training method: CNN training can be divided into three components, namely, initialization of parameters, loss function, and optimization algorithm. All biases of each feature map or node of the output are set to 0, and all weights are initialized by Xavier [42]:

4 $${\text{W}} = \left( {{\text{random}}\left( {0,1} \right) - 0.5} \right) \times 2 \times \sqrt {\frac{6}{neuron\_in + neuron\_out}}$$

where random (0, 1) generates a random number between (0, 1). *neuron*_*in* and *neuron*_*out* are the number of neurons in the upper and next layers, respectively. For the loss function and optimization algorithm, the former represents the dissimilarity of the approximated output distribution from the actual distribution of labels, whereas the latter minimizes this function to improve classifier performance. In our work, a residual learning [43] formulation is adopted to train the network. Loss function is defined as ${\mathcal{L}}\left( \varTheta \right) = \frac{1}{2N}\mathop \sum \nolimits_{i = 1}^{N} \parallel R\left( {p_{i}^{i} ;\varTheta } \right) - \left( {p_{i}^{i} - p_{i}^{f} } \right)\parallel_{F}^{2}$, where (*p*_*i*_^*i*^, *p*_*i*_^*f*^) represents N artifact free-insert training image (patch) pairs. ‖ · ‖_*F*_ is the Frobenius norm. Parameter $\varTheta$ refers to learning from the trainable RL-ARCNN. Figure 2 depicts the workflow of deep CNN for learning consisting of an input layer, an output layer, and convolutional layers.

Adam’s optimization algorithm [44] dynamically updates the learning rate of parameters using the unbiased estimation of the gradient’s first and second moments during backward propagation. This method is used to minimize the loss function in this study.

### RL-ARCNN for MAR

This subsection discusses how the model has been trained to remove metal artifacts. The trained model can predict artifact-residual images (*I*_*artifact*-*residual*_). We then obtain the following:

5 $$I_{artifact - free} = I_{artifact - insert} - I_{artifact - residual}$$

where *I*_*artifact*-*free*_ represents the artifact-free image, *I*_*artifact*-*insert*_ refers to the metal artifact image, and *I*_*artifact*-*residual*_ corresponds to the residual image between the metal artifact and artifact-free images. Based on the aforementioned analysis, the proposed algorithm can be summarized as in Algorithm 1.
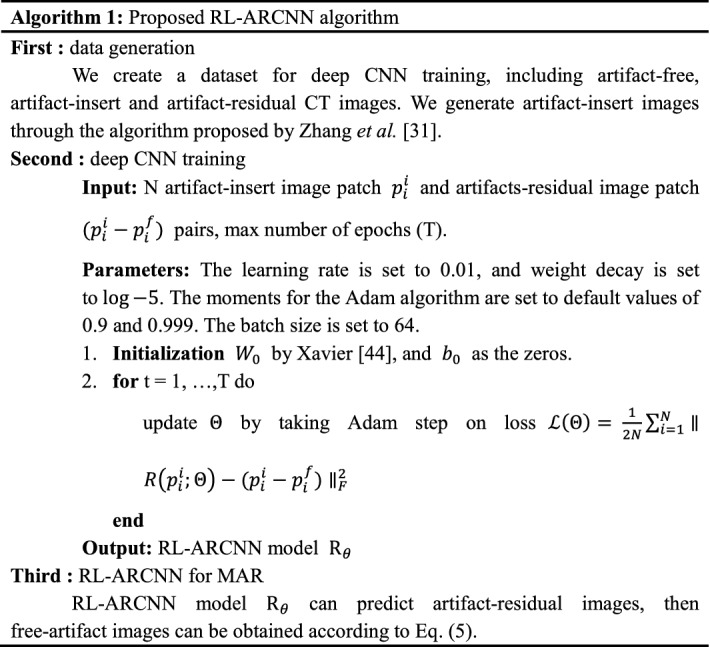


## Experiment results

### Datasets

The simulated artifact cervical CT images and clinical artifact cervical CT images are studied to assess and compare the performances of our proposed methods quantitatively. 35 cervical cancer patients from department of radiotherapy of Nanfang Hospital in Southern Medical University with CT scans before and after brachytherapy were included in this study. This study was approved by the Institutional Review Board, and written informed consent requirement was waived. The simulated artifact cervical CT images (600 slices) were generated from artifact-free CT images of 20 cervical cancer patients before brachytherapy. The clinical artifact cervical CT images (450 slices) were obtained from 15 patients after brachytherapy.

All CT scans were conducted for clinical indications. The CT acquisitions were performed on a Brilliance Big Bore CT scanner (Philips) with the following settings: 120-kVp tube voltage, 375-mA tube current, 3-mm slice thickness, and 512 × 512 imaging matrix that results in an in-plane resolution of 0.738–1.084 × 0.738–1.084.

### Implementation details

#### Training phase

The simulated artifact cervical CT images are used to train and verify our proposed method. Total 600 simulated artifact cervical CT images are divided into three parts, train set (450 slices), validation set (100 slices), and test set (50 slices). The train and validation set are used during training, in which the train set is used to adjust the weights on the neural network, and the validation set is used to minimize overfitting. The test set is used for examining the final solution to confirm the actual predictive power of the network. Small patches are randomly sampled over the entire training set to include numerous images in the single-batch training process. The patch size for model training is 50 × 50. The learning rate is set to 0.01, and weight decay is set to ${\text{log}} - 5$. The moments for the Adam algorithm are set to default values of 0.9 and 0.999. The batch size is set to 64 to completely utilize the GPU memory. A small convolution kernel size of 3 × 3 is adopted for all networks. The model training is repeated for 100 epochs.

The configuration of the computer platform is as follows: CPU of Intel(R) Core(TM) i5-6500 k 3.20 GHz, and NVIDIA TITANX Pascal GPU with 12 G memory. MatConvNet deep learning framework [45] and MATLAB version R2014b are used in this system.

### Results

#### Metric

For quantitative evaluation, the peak signal-to-noise ratio (PSNR) [46] is used, as defined below:

6 $$MSE = \frac{1}{H \times W}\mathop \sum \limits_{i = 1}^{H} \mathop \sum \limits_{j = 1}^{W} \left( {X\left( {i,j} \right) - Y\left( {i,j} \right)} \right)^{2} ,\quad PNSR = 10\log_{10} \frac{{\left( {2^{n} - 1} \right)^{2} }}{MSE}$$

The MSE indicates that the mean square error of the image X and the image Y, H, W indicates the width and height of the images, and n is the number of bits per pixel, which is generally 8. The unit of PSNR is dB and PSNR is based on the errors between the corresponding pixels, that is, the error-sensitive image quality evaluation; a high value indicates good image quality.

#### Evaluation on network setting

(*a) Patch size* We conducted experiments with different image patch sizes and Table 2 shows the evaluation results of different patch size as 25 × 25, 50 × 50, 100 × 100. The results showed that the average PSNR in 50 × 50 patch sizes is 38.09, which provides the best result among three different patch sizes. Furthermore, the influence of residual learning is investigated. Table 3 shows the results of residual learning and ordinary learning. Ordinary learning indicates that a network label is an artifact-free image rather than a residual image. The results also show that CNN-based residual images achieve favorable artifact reduction.

**Table 2:** Quantitative image quality evaluations of the residual learning network based on different input image patch sizes (unit: dB)

Image patch size	25 × 25	50 × 50	100 × 100
PSNR	33.83	38.09	36.80

**Table 3:** Quantitative image quality evaluations with and without residual learning using PSNR (unit: dB)

Image patch size	Artifact-insert	Ordinary-learning	Residual learning
25 × 25	25.38	33.71	33.83
50 × 50	25.38	37.79	38.09
100 × 100	25.38	36.68	36.80

**Fig. 3:**
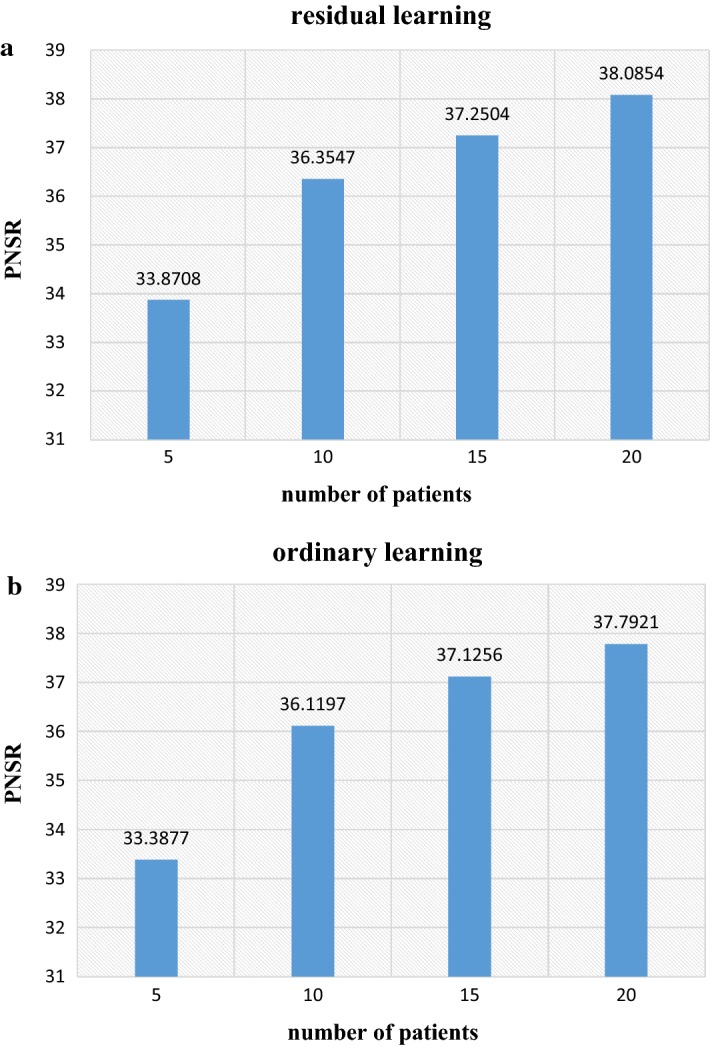
**a** The average PNSR values of using the residual learning network trained with 5, 10, 15 and 20 patients. **b** The average PNSR values of using the ordinary learning network trained with 5, 10, 15 and 20 patients

*(b) Number of training images* We compare the network trained with different numbers of patients. Figure 3a presents average PNSR values of using the residual learning network trained with 5, 10, 15 and 20 patients and the average PNSR values of using ordinary learning network trained with 5, 10, 15 and 20 patients are presented in Fig. 3b. It is clear that the PNSR increases dramatically by applying more training data. This suggests that the performance of the method strongly depends on the size of training data.

**Fig. 4:**
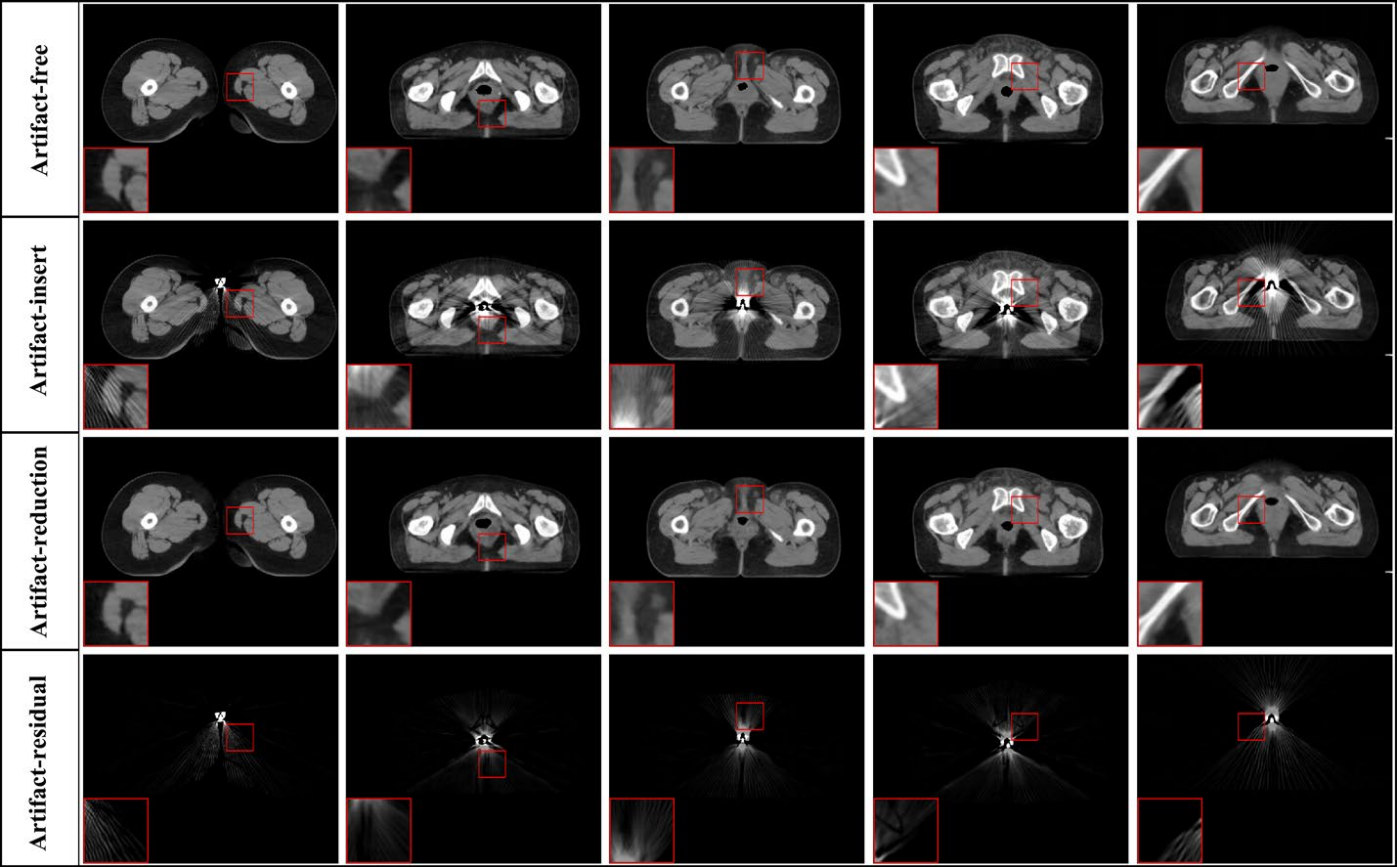
The result of metal artifacts reduction for the simulation data. Each column corresponds one case. The four rows are artifact-insert images, artifact-reduction images, artifact-free and artifact-residual images, respectively. The artifact-residual images are learn from the RL-ARCNN model. The large red box in the lower left corner of each image is the enlarged area of the small red box of the image

*(c) Visual results* The examples of artifact-free, artifact-insert, artifact-reduction, and artifact-residual images are shown in Fig. 4. The artifact-insert images contain metal artifacts that are numerically simulated; the artifact-residual images are learned from the proposed model, whereas the artifact-reduction images are obtained by subtracting the artifacts from artifact-insert images. As shown in Fig. 4, the artifacts are nearly removed completely, and tissue features in the vicinity of metals are authentically preserved.

#### Application of clinical data

**Fig. 5:**
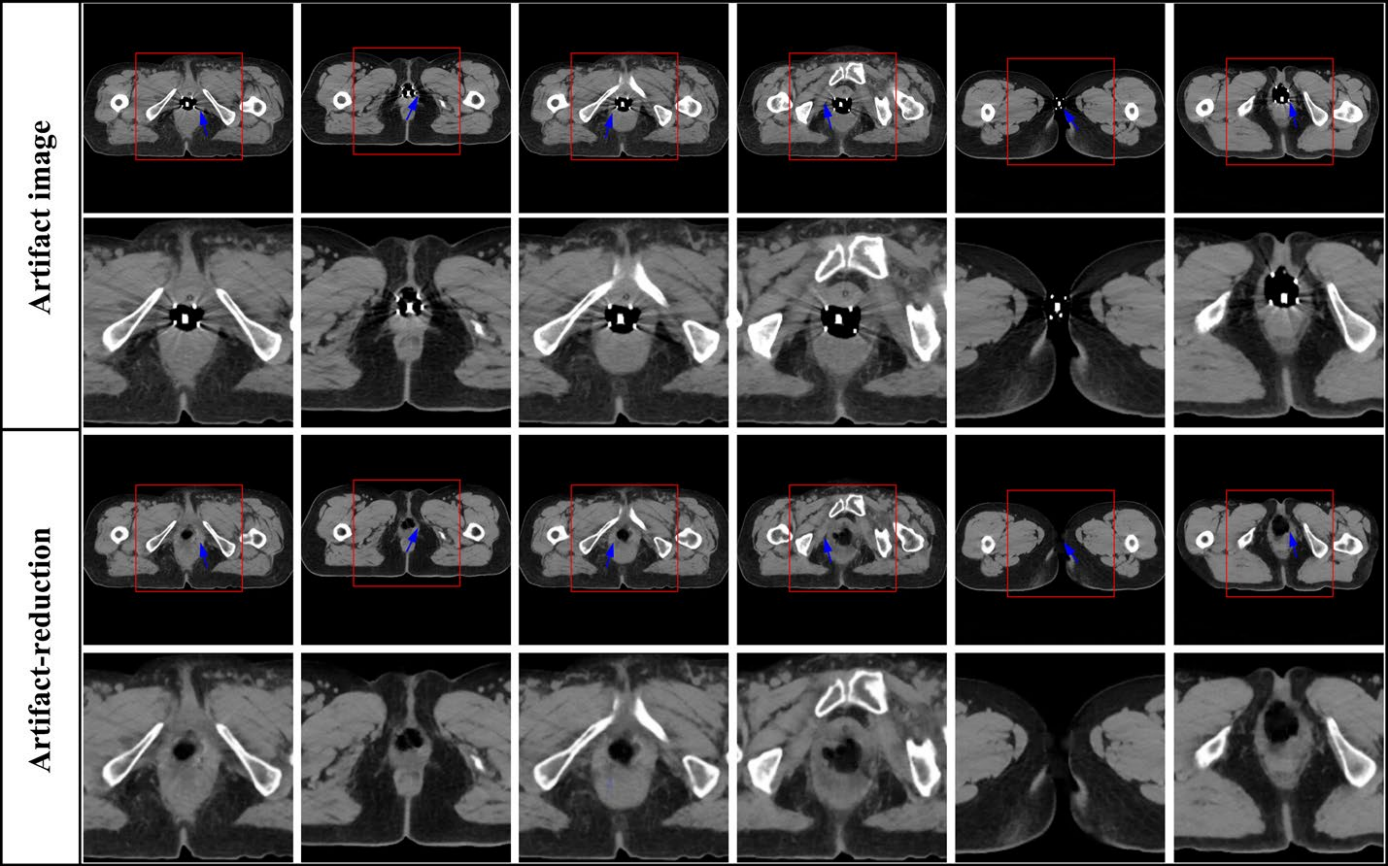
The result of metal artifacts reduction for the clinical artifact cervical CT image caused by interstitial brachytherapy. The first row is CT images with metal artifacts, the third row is metal artifacts reduction images by using the proposed method. The second and fourth row correspond to the enlarged area of the red boxes in the first and third row

**Fig. 6:**
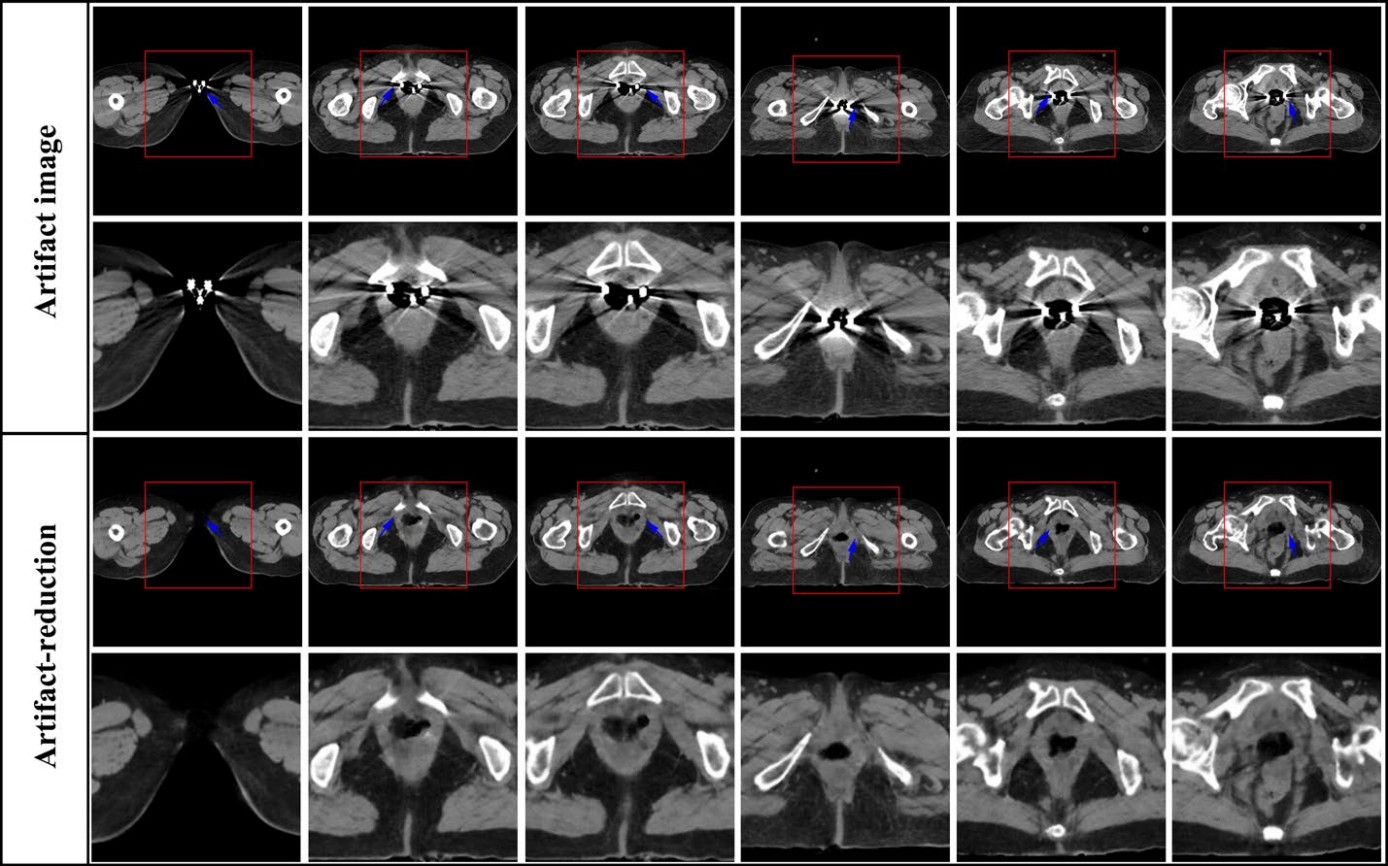
The result of metal artifacts reduction for the clinical artifact cervical CT image caused by intracavitary brachytherapy. The first row is CT images with large metal artifacts, the third row is metal artifacts reduction images by using the proposed method. The second and fourth row correspond to the enlarged area of the red boxes in the first and third row

**Fig. 7:**
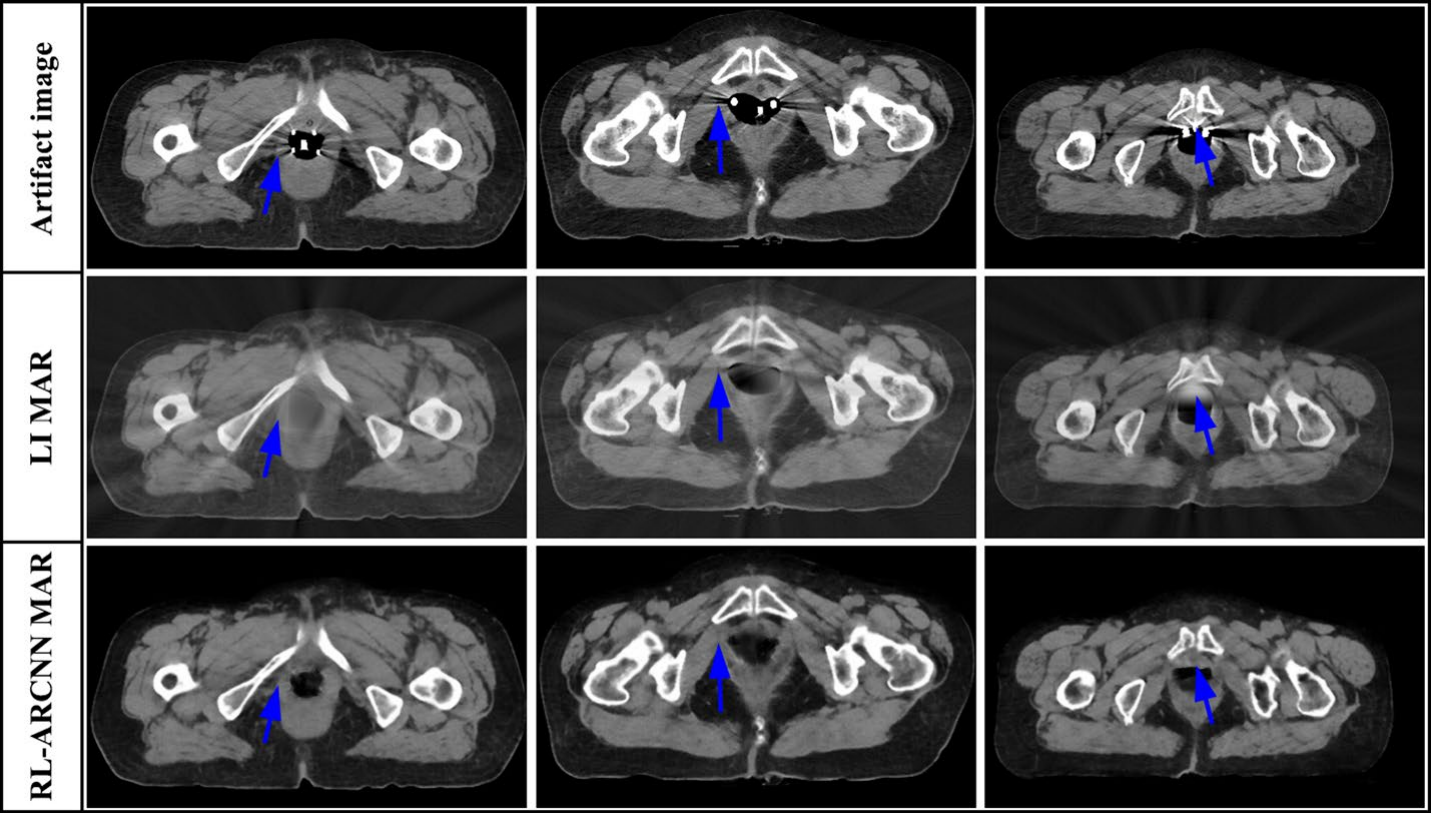
Visual comparison between the MAR results of RL-ARCNN and LI method. The first row is CT images with metal artifacts, and the second and third row are the metal artifacts reduction images by the LI and RL-ARCNN, respectively

The 450 artifact images from 15 patients are employed to evaluate the effectiveness of artifact reduction of clinical images. Since there is no reference image corresponding to the actual clinical metal artifacts images, it is hard to quantitative evaluation of the MAR effects. Figure 5 shows the MAR results on the clinical artifact cervical CT images caused by interstitial brachytherapy. As can be seen from Fig. 5, there are the artifact CT images and enlarged views of selected domains, respectively, in the first and second row. Obvious metal artifacts exist in these images which obscure crucial diagnostic information of tissues surrounding implants, which is indicated by the arrows. By utilizing RL-ARCNN, metal artifacts are efficiently eliminated from CT images to afford images with rather high quality which is show in the third and fourth row in Fig. 5. The results demonstrate the potential application of CNN in the field of MAR. Moreover, RL-ARCNN also expresses sufficient capability to overcome more serious metal artifacts. As shown in Fig. 6, artifact CT images suffer from extremely severe metal artifacts (caused by intracavitary brachytherapy) in the first two rows as indicated by the arrows. Interestingly, clean images with high quality are outputted via our proposed method based on CNN in the lateral two rows as indicated by the arrows. All the artifacts are removed and the crucial tissue information is clearly presented into our view. As shown in Fig. 7, the proposed method effectively reduces metal artifacts, while the LI method is not very clean to remove metal artifacts and creates new streaking artifacts as indicated by the arrows due to the inherent nature of its interpolation technique [9]. Above all, these results indicate that our proposed method is effective and useful in the MAR application.

## Discussion

In this paper, we have proposed a novel residual learning method based on CNN to reduce the metal artifact in cervical CT images for brachytherapy. In order to train RL-ARCNN, a simulated dataset including artifact-insert, artifact-free, and artifact-residual images is generated. Specially, we perform our own simulation data diversification and use different parameters to reconstruct artifact-insert CT images, which are conducive to removal of actual data artifacts. Once the network is trained, RL-ARCNN can quickly remove artifacts from the cervical CT image. For a 512 × 512 image, the average removal artifact time is less than 1 s. Experimental results on simulated dataset demonstrated that proposed CNN model (RL-ARCNN) could nicely remove metal artifact. In addition, experimental results on clinical artifact cervical CT images showed both the effectiveness and robustness of our proposed method.

The key factors to ensure outstanding performance of the RL-ARCNN are two aspects. First, the training performance of artifact-reduced image is stabilized and enhanced by using of batch normalization approach. Second, instead of directly outputting the artifact-reduced image, the proposed RL-ARCNN is designed to predict the residual image, i.e., the difference between the artifact observation and the latent clean image. The experiment results demonstrate that residual learning is effective in boosting the artifact-reduction performance.

The RL-ARCNN offer improved approach to clinical artifact reduction in the imaging routine, as it may allow for a more precise brachytherapy planning. Further, RL-ARCNN can quickly remove artifacts from an artifact cervical CT image. For a 512 × 512 image, the average removal artifact time is less than 1 s and no any post-processing is required, which is suitable for clinical workflows. What’s more, RL-ARCNN does not require special CT scan design, nor does it require projection raw data.

Nevertheless, the proposed method could be further improved from two aspects. First, the capability of the proposed method would increase with increasing training data. Second, the introduction of tissue prior information may help reduce the artifacts further. In the future, we will increase the training data and introduce tissue prior information in the RL-ARCNN framework to improve its capability.

## Conclusions

In this study, we have proposed a deep CNN for metal artifacts reduction, where residual learning is adopted to separate artifacts from metal artifacts images. By applying the designed batch normalization and residual learning, it can accelerate the training process and improve the ability of the CNN for metal artifacts reduction. Both numerical simulations and clinical application have demonstrated that the RL-ARCNN can significantly reduce metal artifacts and restore fine structures near the metals. Because of the data-driven manner of how our deep learning based approach learns features for artifact reduction, it could be generalizable to other artifacts reduction problems.

## Abbreviations

CT: computed tomography
CNN: convolutional neural network
MAR: metal artifact reduction
RL-ARCNN: deep residual learning artifact reduction based on CNN
EM: expectation maximization
ART: algebraic reconstruction technique
ReLU: rectified linear unit
PNSR: peak signal-to-noise ratio
FBP: filtered back projection
